# Efficacy of epidural blood patching or surgery in spontaneous intracranial hypotension: an evidence map protocol

**DOI:** 10.1186/s13643-022-01989-2

**Published:** 2022-06-07

**Authors:** Timothy J. Amrhein, Peter G. Kranz, Sarah Cantrell, Constance R. Deline, Carrie M. Carr, Dong Kun Kim, Karen M. Goldstein, John W. Williams

**Affiliations:** 1 Department of Radiology, Duke University Medical Center, Durham, North Carolina USA; 2 Duke University Medical Center Library, Duke University School of Medicine, Durham, North Carolina USA; 3 Spinal CSF Leak Foundation, Spokane, Washington USA; 4 Department of Radiology, Mayo Clinic, Rochester, Minnesota USA; 5 Division of General Internal Medicine, Duke University School of Medicine, Durham, North Carolina USA; 6 Durham Center of Innovation to Accelerate Discovery and Practice Transformation, Durham Veterans Affairs Health Care System, Durham, North Carolina USA

**Keywords:** Cerebrospinal fluid leak, Epidural blood patch, Spontaneous intracranial hypotension, Surgery

## Abstract

**Background:**

Spontaneous intracranial hypotension (SIH) is a debilitating disorder caused by non-iatrogenic spinal cerebrospinal fluid leaks. SIH is increasingly recognized as an important treatable cause of secondary headaches. Treatment involves either epidural blood patching or surgery, which have considerable differences in their adverse event rates, recovery times, and cost. The objective of this evidence map is to understand the breadth of studies that investigate SIH treatment efficacy and to identify knowledge gaps to inform future research.

**Methods:**

This review will consider experimental, observational, and systematic review studies that assess the efficacy of epidural blood patching and surgery for the treatment of patients with SIH. Individual case studies, clinical guidelines, editorials, protocols, and studies that do not assess an intervention will not be included. English language studies will be included without limitation based on the date of publication. Databases to be searched include MEDLINE® (via Ovid), EMBASE (via Elsevier), and Web of Science™ (via Clarivate). Study selection will be performed independently by two investigators with extracted data to include study type, the number of patients included, patient descriptors, intervention characteristics, and outcome measure used. Data will be presented through a narrative summary aided by tabular and graphical formats in a manner that aligns with the objective of the evidence mapping review.

**Discussion:**

The overarching goal of this evidence map is to provide an improved understanding of the breadth of studies investigating SIH treatment efficacy in the literature and to thereby identify knowledge gaps that can inform future research directions.

**Trial registration:**

OSF Registry https://osf.io/nwju7.

**Supplementary Information:**

The online version contains supplementary material available at 10.1186/s13643-022-01989-2.

## Background

Spontaneous intracranial hypotension (SIH) is a disabling disorder caused by spinal cerebrospinal fluid (CSF) leaks [1, 2]. These CSF leaks are not iatrogenic (e.g., not after a lumbar puncture) and result in debilitating symptoms, most commonly orthostatic headaches [3]. In addition to headaches, patients also may suffer from nausea, cognitive dysfunction, neck and interscapular pain, and cranial nerve symptoms including diplopia, hearing changes, disequilibrium, neuropathies, dementia-like syndromes, and in some cases even coma or death [4]. As a result, many SIH patients experience significant disability leading to reduced work productivity and quality of life.

SIH was once assumed rare but is now recognized as an important treatable cause of secondary headaches. Unfortunately, misdiagnosis and delayed diagnosis remain the norm for SIH patients, with up to 94% experiencing delays in their diagnosis ranging from a month to years [5]. While the prevalence of SIH is largely unknown, one study identified an incidence of 5 in 100,000 in patients that presented to the emergency department at a major urban hospital, which is similar to the rate of better-known causes of secondary headache including subarachnoid hemorrhage [6, 7]. The true incidence is likely much greater due to pervasive misdiagnosis.

Once the diagnosis of SIH is made, efforts turn toward identification and localization of the spinal CSF leak [8]. This then allows for directed treatment in the form of either surgery or epidural blood patching (EBP). EBP is widely accepted as the first-line treatment for SIH and involves the percutaneous injection of autologous sterile blood through a needle into the epidural space. There are several variations of the EBP procedure commonly employed: (a) imaging-guided or non-imaging-guided, (b) targeted directly to the location of a known CSF leak or non-targeted (i.e., patching material placed in the epidural space without regard to leak location), and (c) with or without fibrin glue sealant [9]. The inclusion of fibrin sealant in EBP is an off-label use of this product. Surgical techniques for treatment of SIH include ligation or ablation of a CSF to venous fistula associated with a nerve root sleeve as well as direct repair of the dura via extradural or intradural approaches [10–13]. While EBP is typically the first treatment attempted and effective, with a wide range of reported efficacy (36–90%), many patients require more than one patch [14–21]. Anecdotally, surgery is considered more definitive [10, 12]. However, surgery carries with it an increased risk profile compared with EBP, which has a generally low rate of adverse events, in addition to significantly longer patient recovery times [22]. Further, surgery is considerably more expensive than EBP with total costs often 3–10 times that of patching. For these reasons, there remains equipoise regarding the appropriate treatment algorithm.

We will apply systematic evidence mapping techniques to describe the quantity and types of research studies in the literature that address the efficacy of two treatments for patients with spontaneous intracranial hypertension (SIH), epidural blood patching (EBP), and surgery [23]. We intend for this to be broad in scope and will include patching procedures that are both targeted and non-targeted, imaging-guided and non-imaging guided, and that use blood alone, fibrin glue alone, or a combination of blood and fibrin glue. For surgery, the scope will be inclusive of all types of surgical procedures used to treat spinal CSF leaks and CSF to venous fistulas (CVFs) in patients with SIH. We will also describe any outcome measures used to define the successful treatment of SIH which we envision may include patient symptom response, quality of life or functional status assessments, and imaging biomarkers. Both the use of non-validated assessments (e.g., subjective improvement of headache or general symptoms) and validated outcome measures (e.g., NRS, HIT-6, MIDAS, EQ-5D, PGIC) will be documented. A preliminary search of PROSPERO, MEDLINE, the Cochrane Database of Systematic Reviews, and the *JBI Evidence Synthesis* was conducted revealing only a single published systematic review studying SIH [24]. This project was more broad in scope with the intention to evaluate the clinical characteristics, methods of diagnosis, and general treatment approaches in SIH. Therefore, it does not have a significant overlap with the goals of our evidence map. No other current or underway evidence mapping reviews, scoping reviews, or systematic reviews on the topic were identified. The overarching goal of this evidence map is to provide an improved understanding of the breadth of studies investigating SIH treatment efficacy in the literature and to thereby identify knowledge gaps that can inform future research directions.

## Methods/design

### Review questions/objectives

Two main objectives will be addressed in this evidence map:

i) To describe the published literature that evaluates the efficacy of epidural patching for the treatment of patients with spontaneous intracranial hypotension (SIH).
ii) To describe the published literature that evaluates the efficacy of surgery for the treatment of patients with SIH.

### Inclusion criteria

#### Participants

This review will consider studies that include patients of any age or sex with a reported diagnosis of SIH that were treated with any form of either EBP or surgery. We will also specifically focus on the subset of these studies that include patients that definitively meet the International Classification of Headache Disorders 3rd Edition definition of SIH [25].

Exclusion criteria will be studies of patients with headaches not secondary to SIH, iatrogenic spinal CSF leaks (e.g., post-surgical or due to lumbar puncture), cranial CSF leaks, and mixed populations of SIH and non-SIH patients without subgroup analysis with results reported separately by patients with SIH.

#### Concept

This review will consider studies that assess the efficacy of treatments for SIH, specifically EBP or surgery, and report patient outcomes. Outcomes of interest include patient symptom response, quality of life or functional status assessments, and imaging biomarkers. This includes both non-validated assessments (e.g., subjective improvement of headache or general symptoms) and validated outcome measures (e.g., NRS, HIT-6, MIDAS, EQ-5D, PGIC).

#### Context

This review will consider studies regardless of their location, country, or setting.

#### Types of sources

This evidence mapping review will consider experimental, observational, and systematic review studies that assess the efficacy of epidural blood patching and surgery for the treatment of patients with SIH. This will include randomized controlled trials, non-randomized controlled trials, controlled before and after studies, interrupted time-series, case series, case-control, prospective cohort, retrospective cohort, and relevant systematic reviews. Individual case studies, clinical guidelines, editorials, protocols, and studies that do not assess an intervention will not be included. Only studies published in English will be included without limitation on the date of publication. Publications identified through our search will be used as sources to hand-search references and the gray literature.

#### Search strategy

The search strategy will aim to identify published experimental, observational, and systematic review studies meeting inclusion criteria. The search strategy was developed by the research team and an experienced medical librarian. An initial limited search was developed for MEDLINE (via Ovid); relevant articles from this search, as well as exemplar publications, were mined for keywords. The search strategy utilizes a combination of keywords searched in the titles and abstracts as well as database-specific subject headings. A complete, reproducible search strategy for MEDLINE® (via Ovid) from 1946 to November 20, 2020, is included in Additional file 1. This search strategy will be adapted for each information source. Databases to be searched include MEDLINE® (via Ovid), Web of Science™ (via Clarivate), and EMBASE (via Elsevier). The complete, reproducible search strategies for each database will be included in the final manuscript. Reference lists for all articles included in this evidence map will be reviewed to identify additional relevant publications. The investigators intend to contact the authors of included primary studies for additional information, as needed. Studies published in English will be included, and there will be no exclusion based on the date of publication. To improve specificity, the updated search will exclude animal-only studies. The strategies will be peer-reviewed by a second experienced medical librarian prior to execution using the PRESS Checklist [26].

#### Initial screening search

The established complete search strategy results in a considerable difference in yield with and without the inclusion of terms for the “spontaneous” concept (4995 vs. 820 publications, respectively). As a result, we will begin by reviewing a randomly selected sample of the additional publications that resulted in the larger data set (the set that includes the “spontaneous” concept). Randomized selection will be performed using a random number generator. These additional studies will be reviewed to determine if publications that would meet inclusion criteria for this evidence map would inadvertently be excluded should the more stringent search criteria be employed (by excluding the “spontaneous” concept). Using prespecified inclusion/exclusion criteria, the titles and abstracts of these articles will be reviewed independently by two investigators for potential relevance to the review objectives. Conservatively assuming a 50% probability that a single report in this group will meet the criteria for inclusion, a randomly selected sample of 385 articles would provide a 95% confidence level for confirming that no additional articles would be added if the larger data set was used for study selection. If no additional studies are identified in this cohort, the more stringent search criteria (including terms for the “spontaneous” concept) will be employed. If any additional studies are identified, the larger data set will be used for study selection.

#### Study selection

Using prespecified inclusion/exclusion criteria, the titles and abstracts of articles included in existing reviews and identified through our primary search will be reviewed independently by two investigators for potential relevance to the review objectives. Articles included by either investigator as potentially meeting our inclusion criteria will undergo full-text screening. At the full-text screening stage, two independent investigators must agree on a final inclusion/exclusion decision. Disagreements will be resolved by obtaining a third investigator’s opinion when consensus cannot be reached between the first and second investigators. Reasons for the exclusion of full-text articles will be recorded and reported in the review. Articles meeting eligibility criteria will be included for data abstraction. At full-text review, we will include studies meeting the listed definition of SIH and a treatment intervention (i.e., EPB or surgery), as previously defined. All results will be tracked in both Covidence, a web-based data synthesis software program (Evidence Partners Inc., Manotick, ON, Canada), and EndNote® reference management software (Clarivate) and duplicates removed. Our results will be reported in full in a manner that adheres to Preferred Reporting Items for Systematic Reviews and Meta-Analyses Extension for Scoping Reviews (PRISMA-ScR) guidelines (Additional file 2) [27].

#### Data extraction

Data from included publications will be abstracted into a customized Covidence database by one reviewer and over-read by a second reviewer. Disagreements will be resolved by consensus or by obtaining a third reviewer’s opinion when consensus cannot be reached between the first and second reviewers. Data elements include descriptors to assess study type, number of patients included, and outcome measures used.

Key characteristics abstracted will include patient descriptors (e.g., age, sex, race), intervention characteristics (e.g., type of epidural patching—imaging-guided vs. non-imaging guided, targeted vs. non-targeted, blood only vs. fibrin sealant only vs. blood and fibrin sealant; surgery type), comparator (if any), category of outcome measure used (e.g., patient symptom response, quality of life, functional status, imaging biomarker), specific outcome measure used when applicable (e.g., NRS, HIT-6, MIDAS, EQ-5D, PGIC), timing of outcome assessment after the intervention, and study setting (e.g., country). Multiple reports from a single study will be treated as a single data point, prioritizing results based on the most complete and appropriately analyzed data. When critical data are missing or are unclear in published reports, we will request supplemental data from the study authors. A draft extraction tool is provided as Additional file 3. This extraction tool will be modified and revised as necessary during the process, and modifications will be detailed in the final review manuscript.

#### Data analysis and presentation

We will summarize the literature using relevant data abstracted from the eligible studies. Summary tables will describe the key study characteristics of the primary studies: study design, patient demographics, and details of the treatment intervention. Data will be narratively summarized. Data presentations will include tabular and graphical formats, as appropriate, to convey key features of the literature. We will present our analysis as a broad literature map without commenting on the quality of individual studies or the strength of evidence for the key question.

## Discussion

The resultant evidence map produced from this protocol will be the first generated in the field of SIH. We expect that it will provide impactful information about current gaps in knowledge and thereby substantive guidance directing future research and funding decisions. This evidence map may also serve as a roadmap for planned national SIH guideline initiatives.

## Supplementary Information

**Additional file 1.** Search strategy.

**Additional file 2.** PRISMA-P 2015 checklist.

**Additional file 3.** Data extraction instrument.

## Data Availability

Data sharing is not applicable to this article as no datasets were generated or analyzed during the current study.
