# Supplementary material for: Efficacy of epidural blood patching or surgery in spontaneous intracranial hypotension: an evidence map protocol
Source: Syst Rev. 2022 Jun 7;11:116. doi: 10.1186/s13643-022-01989-2 (PMC9171943; doi:10.1186/s13643-022-01989-2)
Supplement: Supplementary file 1 — Additional file 1. Search strategy. [file 13643_2022_1989_MOESM1_ESM.docx]

Additional file 1: Search Strategy

**Librarian:** Sarah Cantrell, MLIS; Duke University Medical Center Library & Archives, Duke University School of Medicine

**Peer review of search conducted by:** Samantha Kaplan, PhD, MLIS; Duke University Medical Center Library & Archives, Duke University School of Medicine

**BROAD search (does not restrict to *spontaneous*)**

**Database: MEDLINE (via Ovid ALL 1946 to November 20, 2020)**Search date: 11/22/2020

| **Search set** | **Search strategy** | **Results** |
| --- | --- | --- |
| 1  *Intracranial hypotension terms* | exp Intracranial hypotension/ or (intracranial adj3 hypotension).ti,ab,kw. | 1961 |
| 2  *CSF leak terms* | exp Cerebrospinal Fluid Leak/ or ((CSF or cerebrospinal or craniospinal) adj3 (leak or leaks or leaked or leaking or leakage or leakages or hypovolemia or hypovolaemia or hypovolemic or hypovolaemic)).ti,ab,kw. | 9942 |
| 3  *CSF terms* | (low and CSF and volume and headache*).ti,ab,kw. | 41 |
| 4  *CSF terms* | (low and cerebrospinal and fluid and volume and ("head ache*" or headache*)).ti,ab,kw. | 35 |
| 5  *CSF terms* | (low and craniospinal and fluid and volume and ("head ache*" or headache*)).ti,ab,kw. | 2 |
| 6  *Low pressure headache terms* | (low and pressure and ("head ache*" or headache*)).ti,ab,kw. | 1043 |
| 7  *Hypoliquorrhoeic headache terms* | ("hypoliquorrhoeic headache" or "hypoliquorrhoeic headaches" or "hypoliquorrhoeic head ache" or "hypoliquorrhoeic head aches").ti,ab,kw. | 2 |
| 8  *Combining the population terms with OR* | 1 or 2 or 3 or 4 or 5 or 6 or 7 | 12010 |
| 9  *Patching terms* | exp Blood patch, epidural/ or (blood adj3 patch*).ti,ab,kw. or (fibrin adj3 patch*).ti,ab,kw. or (epidural adj3 patch*).ti,ab,kw. | 2013 |
| 10  *Surgical terms* | exp Surgical Procedures, Operative/ or (surgery or surgeries or surgical or microsurgery or microsurgeries or microsurgical or "micro surgery" or "micro surgeries" or "micro surgical" or microdiscectomy or microdiscectomies or microdiskectomy or microdiskectomies or "micro discectomy" or "micro discectomies" or "micro diskectomy" or "micro diskectomies" or "transdural discectomy" or "transdural discetomies" or "transdural diskectomy" or "transdural diskectomies" or "intradural approach" or "intradural approaches" or "posterior approach" or "posterior approaches" or "spinal cord release" or "spinal cord releases" or operative or operation or operations or operated or perioperative or repair or repairs or repaired or ligation or ligations or ligated or ligate or cauteri?e or cauteri?es or cauteri?ed or cauteri?ation or cauteri?ing or clip or clips or clipped or clipping or coagulation or coagulate or coagulates or coagulated or coagulating).ti,ab,kw. | 4697477 |
| 11  *Combining the intervention terms with OR* | 9 or 10 | 4698486 |
| 12  *Combining the population + intervention terms with AND* | 8 and 11 | 8573 |
| 13  *Excluding animal-only studies* | 12 NOT (exp animals/ NOT exp humans/) | 8482 |
| 14  *Excluding publication types* | 13 NOT (Editorial.pt. OR Letter.pt. OR Case Reports.pt. OR Comment.pt.) | 5789 |
| 15  *Limit to English language* | limit 14 to english language | 4995 |

**NARROW search *(restricts to including terms related to spontaneous)***

**Database: MEDLINE (via Ovid ALL 1946 to November 20, 2020)**Search date: 11/22/2020

| **Search set** | **Search strategy** | **Results** |
| --- | --- | --- |
| 1  *Intracranial hypotension terms* | exp Intracranial hypotension/ or (intracranial adj3 hypotension).ti,ab,kw. | 1961 |
| 2  *CSF leak terms* | exp Cerebrospinal Fluid Leak/ or ((CSF or cerebrospinal or craniospinal) adj3 (leak or leaks or leaked or leaking or leakage or leakages or hypovolemia or hypovolaemia or hypovolemic or hypovolaemic)).ti,ab,kw. | 9942 |
| 3  *CSF terms* | (low and CSF and volume and headache*).ti,ab,kw. | 41 |
| 4  *CSF terms* | (low and cerebrospinal and fluid and volume and ("head ache*" or headache*)).ti,ab,kw. | 35 |
| 5  *CSF terms* | (low and craniospinal and fluid and volume and ("head ache*" or headache*)).ti,ab,kw. | 2 |
| 6  *Low pressure headache terms* | (low and pressure and ("head ache*" or headache*)).ti,ab,kw. | 1043 |
| 7  *Hypoliquorrhoeic headache terms* | ("hypoliquorrhoeic headache" or "hypoliquorrhoeic headaches" or "hypoliquorrhoeic head ache" or "hypoliquorrhoeic head aches").ti,ab,kw. | 2 |
| 8  *Combining the population terms with OR* | 1 or 2 or 3 or 4 or 5 or 6 or 7 | 12010 |
| 9  *Spontaneous terms* | (spontaneous or spontaneously or sudden or suddenly or immediate or immediately or unprompted or unprovoked).ti,ab,kw. | 873403 |
| 10  *Combining population with spontaneous* | 8 and 9 | 2634 |
| 11  *Patching terms* | exp Blood patch, epidural/ or (blood adj3 patch*).ti,ab,kw. or (fibrin adj3 patch*).ti,ab,kw. or (epidural adj3 patch*).ti,ab,kw. | 2013 |
| 12  *Surgical terms* | exp Surgical Procedures, Operative/ or (surgery or surgeries or surgical or microsurgery or microsurgeries or microsurgical or "micro surgery" or "micro surgeries" or "micro surgical" or microdiscectomy or microdiscectomies or microdiskectomy or microdiskectomies or "micro discectomy" or "micro discectomies" or "micro diskectomy" or "micro diskectomies" or "transdural discectomy" or "transdural discetomies" or "transdural diskectomy" or "transdural diskectomies" or "intradural approach" or "intradural approaches" or "posterior approach" or "posterior approaches" or "spinal cord release" or "spinal cord releases" or operative or operation or operations or operated or perioperative or repair or repairs or repaired or ligation or ligations or ligated or ligate or cauteri?e or cauteri?es or cauteri?ed or cauteri?ation or cauteri?ing or clip or clips or clipped or clipping or coagulation or coagulate or coagulates or coagulated or coagulating).ti,ab,kw. | 4697477 |
| 13  *Combining the intervention terms with OR* | 11 or 12 | 4698486 |
| 14  *Combining the population + intervention terms with AND* | 10 and 13 | 1777 |
| 15  *Excluding animal-only studies* | 14 NOT (exp animals/ NOT exp humans/) | 1767 |
| 16  *Excluding publication types* | 15 NOT (Editorial.pt. OR Letter.pt. OR Case Reports.pt. OR Comment.pt.) | 921 |
| 17  *Limit to English language* | limit 16 to english language | 820 |
