# Supplementary material for: Efficacy of epidural blood patching or surgery in spontaneous intracranial hypotension: an evidence map protocol
Source: Syst Rev. 2022 Jun 7;11:116. doi: 10.1186/s13643-022-01989-2 (PMC9171943; doi:10.1186/s13643-022-01989-2)
Supplement: Supplementary file 3 — Additional file 3. Data extraction instrument. [file 13643_2022_1989_MOESM3_ESM.docx]

Additional file 3: Data Extraction Instrument

Study Author / Year of Publication

Study setting: Location: (e.g., country)

Study setting:

- Not specified
- Private practice
- Academic center
- Referral center (i.e. dedicated SIH program)
- Other (free text)

Study Type:

- Systematic review
- Randomized controlled trial
- Non-randomized controlled trial
- Controlled before and after study
- Interrupted time-series
- Prospective cohort
- Retrospective cohort
- Case control
- Cross sectional
- Case series

SIH Diagnosis - Method:

- Method not specified
- Subjective (e.g. reviewed by expert confirming SIH, specific criteria not reported)
- Objective (specific reported criteria: e.g. imaging, CSF pressure, etc.)
  - Meets current ICHD-3 standards
  - Does not meet current ICHD-3 standards
  - Cannot determine if meets ICHD-3 standards
- Additional comments (free text)

Type of CSF Leaks:

- Not specified
- Epidural leak – diverticular
- Epidural leak – osteophyte spur
- CSF to venous fistula
- More than one type

Number of Patients (per subgroup, as appropriate)

Demographics (per subgroup, as appropriate):

- mean age and ranges
- sex (% female)
- race(s) (if available)

Intervention Characteristics:

- Epidural Blood Patch: Y or N
- Epidural Blood Patch type:
  - imaging guided
  - non-imaging guided
  - targeted
  - non-targeted
  - blood only
  - fibrin sealant only
  - blood and fibrin sealant
  - not specified
  - other (free text)
- Surgery: Y or N
- Surgery type:
  - nerve root ligation or clipping
  - electrocautery of epidural veins
  - dural repair: intradural approach
  - dural repair: extradural approach
  - not specified
  - other (free text)

Comparator group: Y or N

- Free text

Outcomes:

- Patient symptom response
  - not reported / not performed
  - reported: subjective (non-validated) assessment
  - reported: validated outcome measures (free text: (e.g. NRS, HIT-6, MIDAS))
- Quality of life or functional status assessments
  - not reported / not performed
  - reported: subjective (non-validated) assessment
  - reported: validated outcome measures (free text: (e.g. EQ-5D))
- Imaging biomarkers
  - not reported / not performed
  - reported: subjective (non-validated) assessment
  - reported: objective
  - reported: validated outcome measures (free text: (e.g. Dobrocky))
- Timing of outcome assessment after intervention
  - not applicable / outcome assessment not performed
  - short term: < 1 month
  - intermediate term: 1 – 6 months
  - long term: > 6 months
  - free text

Additional comments (free text)
